# Use of mycorrhizal fungi and phosphorus fertilization to improve the yield of onion (*Allium cepa* L.) plant

**DOI:** 10.1016/j.sjbs.2021.08.094

**Published:** 2021-09-06

**Authors:** T.M.S. El-Sherbeny, Abeer M. Mousa, El-Sayed R. El-Sayed

**Affiliations:** a Plant Research Department, Nuclear Research Center, Egyptian Atomic Energy Authority, Cairo, Egypt; b Soil and Water Research Department, Nuclear Research Center, Egyptian Atomic Energy Authority, Cairo, Egypt

**Keywords:** Onion, Mycorrhizal Fungi, Economic yield, P fertilization, N utilization, ^15^N

## Abstract

Improving the economical yield of commonly cultivated crops is one of the most pressing social and scientific issues in modern agriculture. This paper was conducted to investigate the bio-efficacy of arbuscular mycorrhizal fungi (AMF) in improving phosphorous (P) utilization and increasing the yield of onion plant grown in sandy soil under a drip irrigation system. The obtained results showed that AMF inoculation of onion and application of 120 kg P fertilizer ha^−1^ significantly increased the fresh and dry weights, chlorophyll content of onion as well as P concentration in the root, shoot, and bulb during two growing seasons. Moreover, AMF increased the bioavailability of P in the rhizosphere and significantly enhanced the N-utilization by the inoculated plant. The economic yield of the onion plant inoculated by AMF and fertilized by different doses of P fertilizer was much higher than that obtained by the control (without AMF). These findings indicated that inoculating the onion plant in the field with AMF could be very effective in increasing the yield of the onion plant. Additionally, this study suggests AMF as a low-cost and promising candidate for the sustainable production of the onion crop using reclaimed sandy soils and a drip irrigation system.

## 1 Introduction

Onion, *Allium cepa* L., is one of the most important commercial crops and ranked as the second economical value after some cultivated vegetables (FAO, 2020). The plant has a distinctive flavor and is used in dishes, soups, sandwiches, and salad and also is cooked alone as a vegetable. Onion is consumed after maturity in the form of a dry-bulb or at its young green stage. The mature dry bulbs contain proteins, starch, sugars, and some vitamins (Jilani et al., 2010). Besides its nutritional value, onion showed some medical applications because of containing several anticancer agents (Bagali et al., 2012) which have been shown to prevent cancer in animals. Generally, the edible parts of *Allium* spp. plants contain volatile sulfur-containing compounds, which have a distinct flavor and are responsible for the pungent odor (Gîtina et al., 2014). Biologically active organo-sulfuric compounds, S-alk(en)yl-L-cysteine sulfoxides (such as alliin and g-glutamylcysteines) dominates onion flavor. In addition, the sulfoxides are found in the cytoplasm and in the vacuole. These compounds are responsible for the characteristic smell and taste of onion and most of their biological properties (Lanzotti 2006).

Currently, chemical fertilizers are used to increase the production of most crops and meet the growing demands for food due to the increasing human population. Unfortunately, these fertilizers had high production costs and showed a harmful effect on the environment. Thus, the interest in finding alternative green sources such as biofertilizers as eco-friendly systems with the ability to improve the crop yield and lower the cost of production is growing and will continue to rise. Recently, the use of microbial populations as bio-fertilizers have emerged as promising alternatives of these chemicals for lower-cost production, enhanced environmental sustainability, and improved yield (Egamberdiyera, 2007).

Several reports revealed that arbuscular mycorrhizal fungi (AMF) can increase both water and nutrient absorption as well as increasing the bioavailability of phosphorus (P) to host and improve plant growth (Golubkina et al., 2020a, and refrences therein). AMF have synergistic effects with other microorganisms and improve the yield of several crops (Lukiwatid and Simanungkalit, 2002, Heggo and Barakah, 2003, Marulanda et al., 2003). Generally, plant and AMF interaction is an important association in the rhizosphere with improvement physical, chemical, and biological properties in soil (Smith and Smith, 2011). AMF operated in rhizosphere soil by using a wide range of different mechanisms. Such nutrients dissolving phosphorus through the production of organic acid (Eldhuset et al., 2007). The use of AMF as phosphate dissolving microorganisms was proposed as a low-energy and low-cost mechanism that enhances the effectiveness of phosphate fertilizers (Richardson, 2001, Gyaneshwar et al., 2002). Nitrogen fertilizer is also responsible for an important part of agriculture-related pollution through nitrate leaching, ammonia volatilization, and nitrous oxide emissions; thus, the efficiency of fertilizer utilization use has to be considered (Lobos Ortega et al., 2016).

In this paper, the role of AMF in reducing phosphorous (P) fertilization, improving nitrogen utilization, and increasing the yield of onion plant grown in sandy soil under a drip irrigation system was assessed for the first time. We also evaluated the rate of root colonization and its potential for improving the P levels in the rhizosphere during the two successive growing seasons.

## 2 Materials and methods

### 2.1 Plant and soil

Onion (*Allium cepa* L., cv. Shandweel) seeds were purchased from the Agronomy Research Institute (Agricultural Research Centre, Giza, Egypt). Field soil in this study was reclaimed from sandy soil. It was subjected to chemical, physical and mechanical analyses (Carter and Gregorich 2007). All the studied parameters are listed in Table 1.

**Table 1:** Mechanical, chemical and physical properties of soil.

Chemical properties		Physical properties		Mechanical properties	
0.91%	Organic matter	17.21%	Field capacity	26.60%	Coarse sand
35 (mg kg^−1^)	Available N	6.07%	Wilting point	52.60%	Fine sand
18 (mg kg^−1^)	Available P	11.22%	Available water	4.30%	Clay
141 (mg kg^−1^)	Available K	1.65 (g cm^−3^)	Bulk density	16.50%	Silt
0.68	EC (ds m^−1^)	7.69	pH (1: 2.5)	Sandy soil	Texture

### 2.2 Isolation arbuscular mycorrhizal fungi (AMF) and inoculum preparation

Spores of AMF were extracted from the rhizosphere of a fertile soil cultivated with onion plants at the Nuclear Research Center, Egyptian Atomic Energy Authority, Inshas, Egypt. The mycorrhizal spores were extracted by the wet sieving and decanting technique according to Gerdemann and Nicolson (1963). The extracted AMF spores were stored at 4 °C until used. Morphological characteristics of attached hyphae, hyphae, azygospores, chlamydospores, and sporocarps of the collected AMF were extensively studied. Identification and characterization were carried out according to the key of Schenck and Perez (1990). Distribution of the extracted AMF genera in 1 kg of soil sample was as follows: *Glomus* sp. 80%, *Gigaspora* sp. 15%, *Acaulospora* sp. 3%, and *Sctellospora* sp. 2%. AMF inoculation was carried out after each season as follows: 100 mL spore suspension (contains 30,000 spores) was added to 1 kg sterilized soil, distributed evenly to the whole plot, mixed by tillage with soil at planting.

### 2.3 Field experiment

Two field experiments were carried at the experimental farm of the Nuclear Research Center (Egyptian Atomic Energy Authority, Inshas, Egypt) during the two growing seasons of 2018/2019 and 2019/2020 to study the effect of arbuscular mycorrhizal fungi (AMF) inoculation interacted with mineral P fertilizer on growth and yield of the onion plant. Onion seeds were planted in a nursery for 55 days and then transplanted to the field. The transplanting was settled upon the two sides of the irrigation line included a dripper 25 cm along. The experimental area was divided into equal size plots; the plot area was (1.5 × 2) 3 m. The experiments were included three levels from monocalcium phosphate fertilizer (15.5% P_2_O_5_) as follows 40, 80, and 120 kg ha^−1^ with or without AMF inoculation (control). One level of nitrogen fertilizer was added at a rate of 140 kg N ha^−1^ in the form of ammonium sulfate (21.2 % N) at two equal doses whereas the first started at 21 days after transplanting and the second started 21 days after the first time. (^15^NH_4_)SO_4_ with enrichment 5 % N atom excess was mixed with ordinary fertilizer in microplot (0.60 m long × 0.50 m apart) to be 0.30 m^2^ for measuring nitrogen efficiency as N-utilized. Potassium sulfate (48% K_2_SO_4_) at the rate of 200 kg ha^−1^ was also applied before transplanting.

### 2.4 Estimation of root colonization

Root samples were collected during the two seasons of the experiment after 30, 60, and 90 days after transplanting. The collected samples were stained according to the method described by Phillips and Hayman (1970). In brief, roots were washed in sterile distilled water, softened for 24 h in KOH (10%), washed in sterile distilled water. After which, roots were acidified for 1 h at room temperature in lactic acid (5%) and then stained for 24 h with aniline blue (0.01%) in pure lactic acid. Finally, roots were cut into 1 cm sections and mounted onto slides in glycerol then analyzed using a computerized light microscope (Motic images 2000, version 1–3, Micro-Optic Industrial Group Co. Ltd, Japan). The percentage of AMF colonization was calculated according to Trouvelot et al. (1986).

### 2.5 Measured plant parameters

Plants samples were taken 110 days after transplanting for estimating both fresh and dry weight (g plant^−1^) and total chlorophyll contents (mg g^−1^ dry weight) in plant leaves according to the method described by Gross (1991). In brief, leaves were ground in cool water in darkness and adjusted to volume 25 mL by distilled water. Then 0.5 mL of this solute was mixed with 4.5 mL acetone 80% and centrifuged 3000 rpm for 10 min. The upper zone of this solution was taken for spectrophotometery (Gross, 1991).

At the harvest time, the economical yield expressed as bulbs weight (kg ha^−1^) was measured. Besides, shoots, roots, bulbs were dried and ground to determine the phosphorous content according to the method described by Cottenie et al. (1982). In brief, plant sample was placed in 100 mL flask and Darco-G-60 was added followed by 50 mL of NaHCo_3_ (0.5 M) solution, next flask was corked, and allowed for shaking for 30 min. The content was then filtered. 5 mL filtrate was taken in 25 mL volumetric flask and 2 drops of 2, 4- paranitrophenol were added. 5 N H_2_SO_4_ drop by drop was added with intermittent shaking till yellow color disappear, content was diluted about 20 mL with distilled water and then 4 mL ascorbic acid was added then the mixture was shacked well and the intensity of blue color at 660 nm on colorimeter was measured. The absorbances were compared and concentrations of phosphorous using standard value were calculated.

### 2.6 Chemical analysis

Rhizosphere soil samples were taken 90 days after transplanting for estimation of available phosphorus in soil according to the method described by Olsen et al. (1954). Total nitrogen determination according to Bremner and Mulvaney (1982). ^15^N percentage in plant sample as atom excess which determined using emission spectrometry as described by Bursh et al. (1982).

### 2.7 Statistical analysis

Experimental results were expressed as the mean of ten replicates. Experiments were designed in a randomized complete block design in ten replicates. The data in each season were statistically analyzed according to procedures outlined by Snedecor and Cochran (1989). The treatment means and interaction were compared using the least significant difference (LSD) test.

## 3 Results

### 3.1 Effect of phosphorus fertilization and AMF inoculation on root colonization

**Fig. 1:**
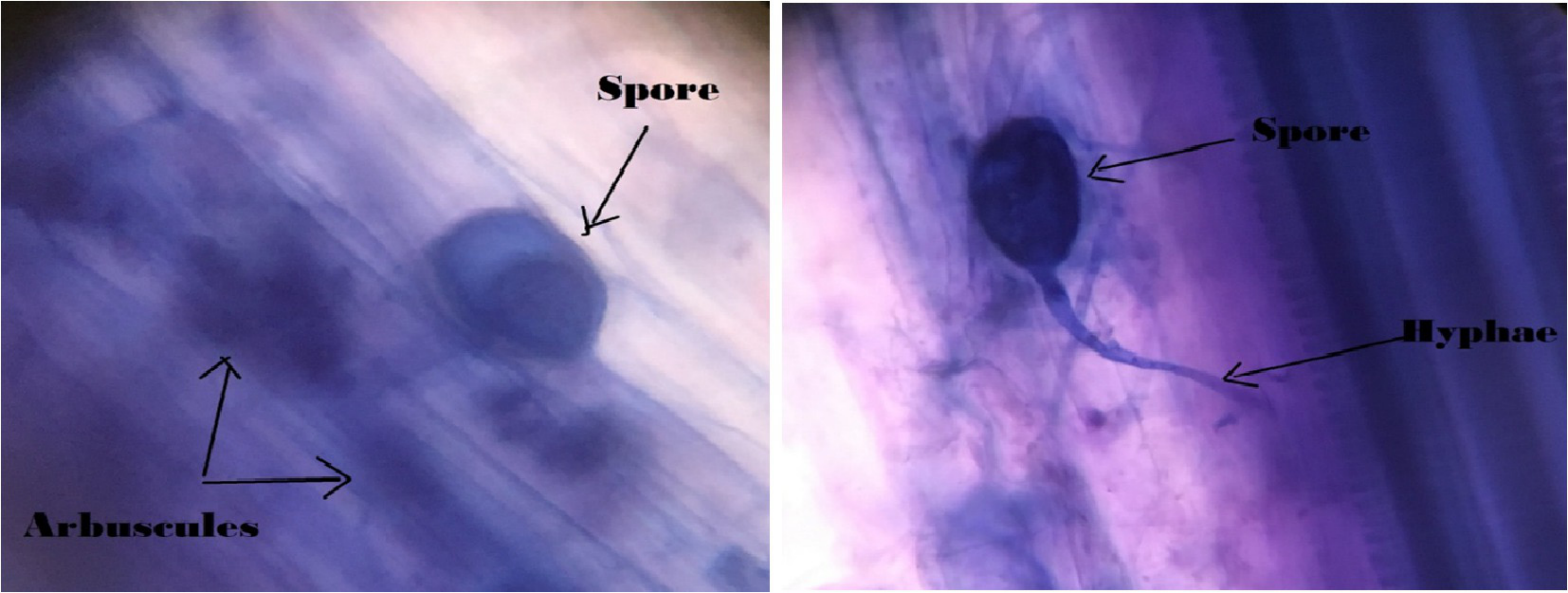
Microscopic visualization (40×) of arbuscular mycorrhizal fungi showing arbuscules and spores **(a)** and spores and hyphae **(b)** in the onion root.

The effect of AMF inoculation on root colonization was recorded after 30, 60, and 90 days of transplanting. Fig. 1 presents the microscopic visualization of AMF showing arbuscules, spores, and hyphae in the roots of onion. The obtained results clearly showed that no colonization on root after 30 days. Results presented in Table 2 confirmed that the colonization of AMF in the roots of onion after 60 days of planting reached 70% with level 80 kg P fertilizer ha^−1^. Similarly, Table 3 showed AMF colonization increased most rapidly reaching 81% after 90 days with level 80 kg P fertilizer ha^−1^ during the two growing seasons. The inoculation by AMF and application of P fertilizer together were more effective in enhancing root colonization. The highest records of AMF infection percentages were recorded using 80 kg ha^−1^ P fertilizer for the onion plant through the two growing seasons. Mycorrhizal colonization of roots was declined when increasing above 80 kg P_2_O_5_ ha^−1^ either at 60 or 90 days (Table 2, Table 3).

**Table 2:** Effect of AMF inoculation on mycorrhizal colonization (%) after 60 days of onion plant growth under different levels of P fertilizer.

P fertilizer conc. (kg ha^−1^)	Mycorrhizal colonization (%)			
	Season 2018/2019		Season 2019/2020	
	Control	AMF	Control	AMF
40	0.001	23.0	9.67	30.33
80	0.001	70.0	10.33	70.33
120	0.001	39.0	10.33	43.66
LSD (0.05)	P fertilizer: 1.72 AMF: 1.41 P fertilizer AMF: 2.43		P fertilizer: 1.40 AMF: 1.15 P fertilizer AMF: 1.98

**Table 3:** Effect of AMF inoculation on mycorrhizal colonization (%) after 90 days of onion plant growth under different levels of P fertilizer.

P fertilizer conc. (kg ha^−1^)	Mycorrhizal colonization (%)			
	Season 2018/2019		Season 2019/2020	
	Control	AMF	Control	AMF
40	0.001	36.33	14.33	45.33
80	0.001	80.66	12.33	81.33
120	0.001	45.66	10.66	53.33
LSD (0.05)	P fertilizer: 0.99 AMF: 0.81 P fertilizer AMF: 1.39		P fertilizer: 1.28 AMF: 1.05 P fertilizer AMF: 1.81	

### 3.2 Effect of phosphorus fertilization AMF inoculation on availability of P in the rhizosphere

Table 4 presented the effect of AMF with different P levels on the availability of P in soil rhizosphere after 90 days from planting. The obtained data (Table 4) indicate that the inoculation with AMF increased P concentration in the soil as compared with control treatment (without AMF). The obtained data further showed that increasing phosphorus fertilization was accompanied by an increase in the recorded P in the rhizosphere. The highest concentrations (54.39% for the season 2018/2019, and 76.13% for the season 2019/2020) of the available P in the rhizosphere during the two growing seasons were recorded at 120 kg P_2_O_5_ ha^−1^. The maximum available P in soil with level 120 kg P_2_O_5_ ha^−1^ was 76.13% compared with control which was 20.20% in the same level in the second season. Similar results with too lower extent were noticed during the first season.

**Table 4:** Effect of AMF inoculation on concentration of the available phosphorus (%) in rhizosphere after 90 days of onion plant growth under different levels of P fertilizer.

P fertilizer conc. (kg ha^−1^)	Concentration of phosphorous (%) in rhizosphere			
	Season 2018/2019		Season 2019/2020	
	Control	AMF	Control	AMF
40	19.60	24.50	20.57	30.43
80	21.33	50.87	25.03	59.17
120	20.30	54.93	20.20	76.13
LSD (0.05)	P fertilizer: 0.64 AMF: 0.52 P fertilizer AMF: 0.92		P fertilizer: 0.37 AMF: 0.30 P fertilizer AMF: 0.53	

### 3.3 Effect of phosphorus fertilization and AMF inoculation on growth parameters of the onion plant

**Fig. 2:**
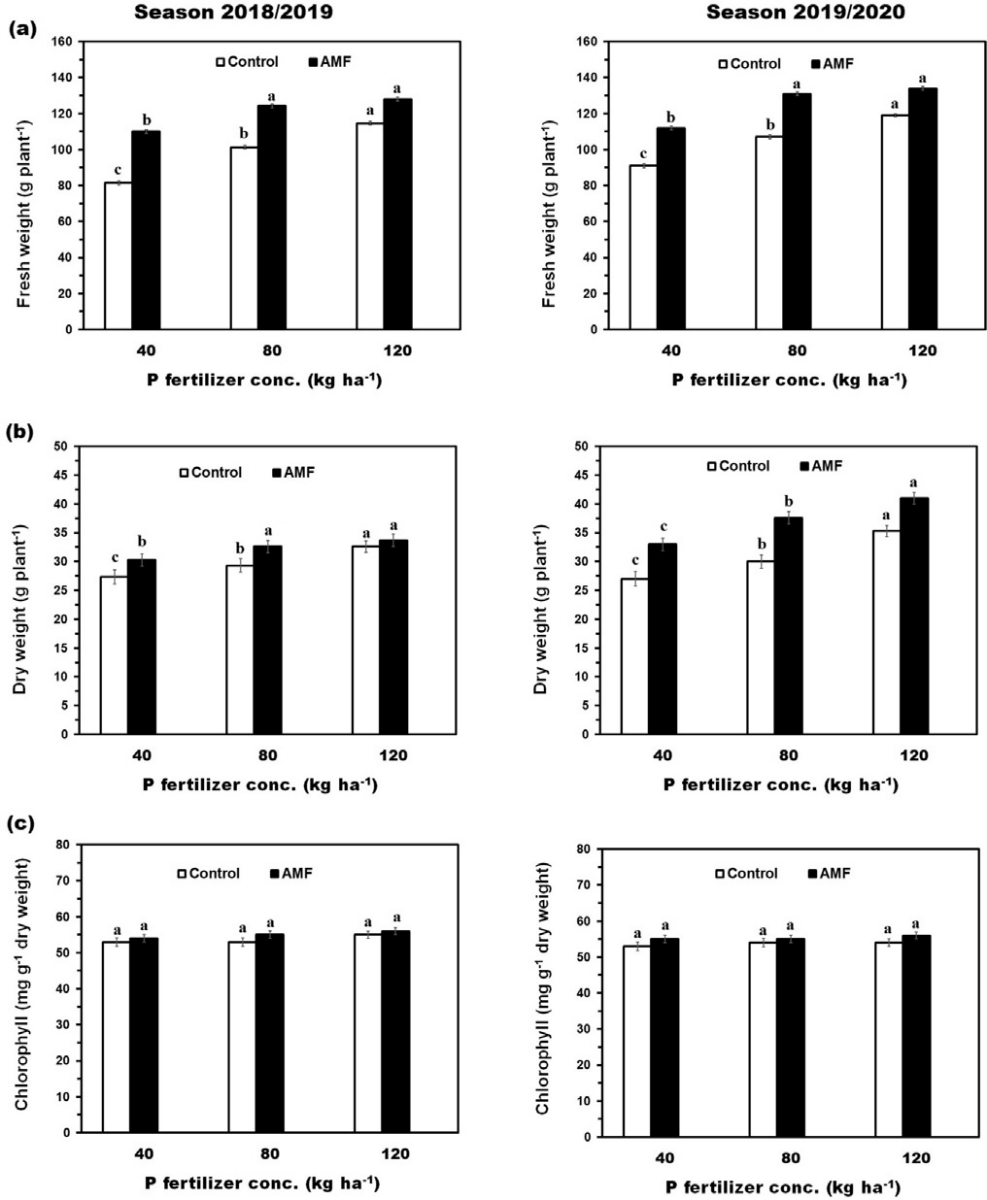
Growth parameters of the onion plant influenced by AMF inoculation and different levels of phosphate fertilizer. **(a)** Effect of AMF inoculation and different levels of phosphate fertilizer on fresh weight (g plant^−1^) of the onion plant. **(b)** Effect of AMF inoculation and different levels of phosphate fertilizer on dry weight (g plant^−1^) of the onion plant. **(c)** Effect of AMF inoculation and different levels of phosphate fertilizer on chlorophyll (mg g^−1^ dry weight) of the onion plant. Data on the left-hand side represent the 1st season and the right-hand side represent the 2nd season. Means with different letters for each group (Control or AMF) were considered statistically different.

Data presented in Fig. 2 clearly show that fresh and dry weight of onion plants positively responded to AMF inoculation with each mineral phosphorus fertilizer level as compared with control (without AMF). The recorded values of fresh and dry weight gave maximum values with mycorrhizal inoculation at 120 kg P_2_O_5_ ha^−1^ as the recommended dose. This observation was obvious little difference values between values of dry and fresh weight with increase phosphorus fertilizer levels between the second level 80 kg P_2_O_5_ ha^−1^ and the third level 120 kg P_2_O_5_ ha^−1^. Relatively, mycorrhizal inoculation caused increments in fresh weight (34.8, 22.7, and 11.7%, for the 1st season and 23.1, 22.4, and 12.6%, for the 2nd season) with mycorrhizal inoculation over the control with the three respective P fertilizer concentrations. The results showed that the dry weight per plant increased by increasing the phosphate fertilizer rate from 40 to 120 kg P_2_O_5_ ha^−1^ in 2018/2019 and 2019/2020 growing seasons as shown in Fig. 2. The highest dry weight was 33.7 and 32.6 in the first season and 41.0 and 35.3 in the second season with or without mycorrhizal inoculation, respectively. The data illustrated in Fig. 2c showed that all different levels of phosphorus fertilization with or without mycorrhizal inoculation affected of total chlorophyll content. The obtained results further showed that chlorophyll content in inoculated plants was slightly higher than in control. Application of phosphorus fertilizer up to 120 kg P_2_O_5_ ha^−1^ with mycorrhizal inoculation gave the maximum mean values of total chlorophyll which equal to 56.2 and 56.0 mg g^−1^ dry weight at 1st and 2nd season, respectively.

### 3.4 Effect of phosphorus fertilization and AMF inoculation on phosphorus concentration in onion plant parts

**Fig. 3:**
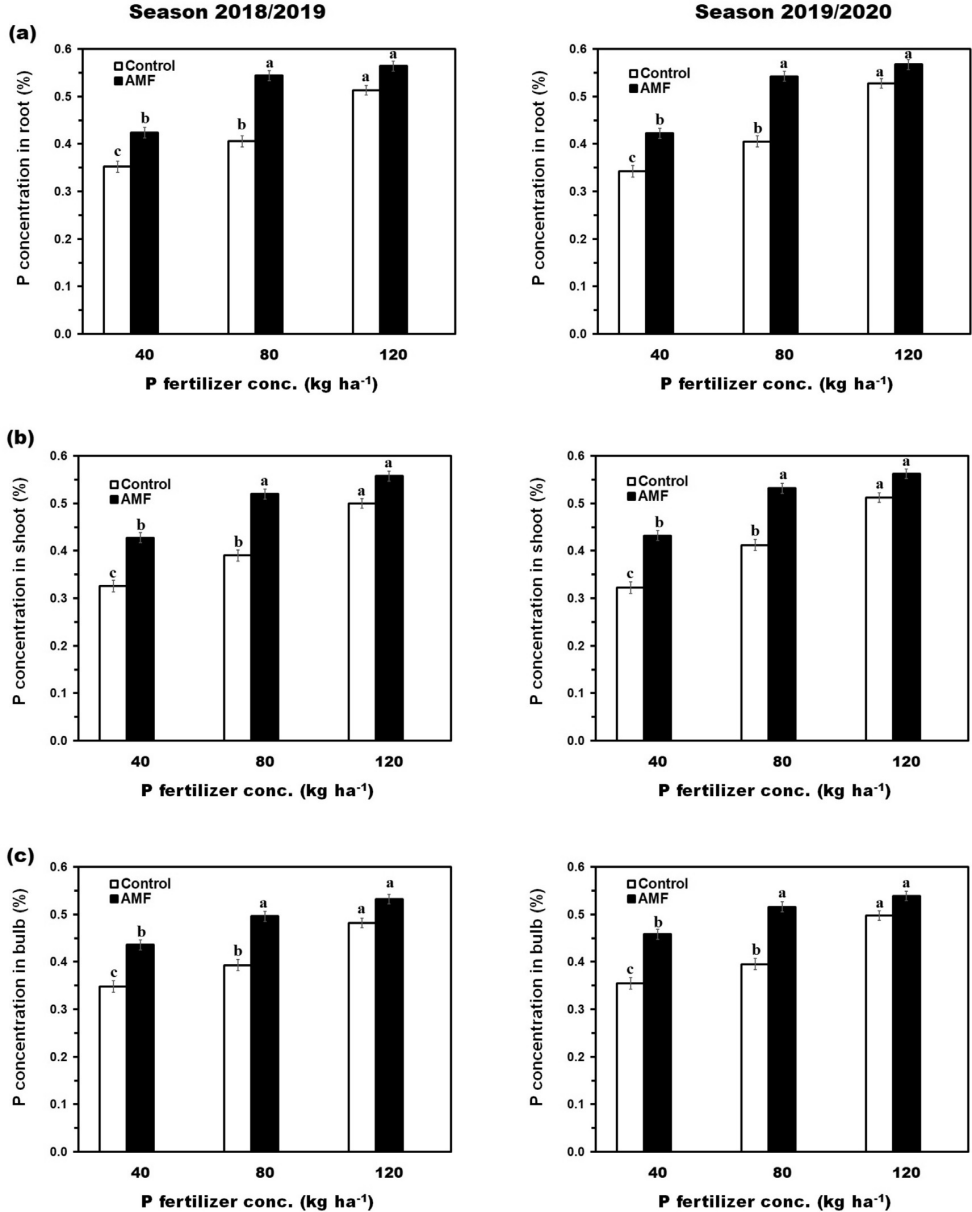
The concentration of P in the onion plant influenced by AMF inoculation and different levels of phosphate fertilizer. **(a)** Effect of AMF inoculation and different levels of phosphate fertilizer on P content (%) in roots of the onion plant. **(b)** Effect of AMF inoculation and different levels of phosphate fertilizer on P content (%) in shoots of the onion plant. **(c)** Effect of AMF inoculation and different levels of phosphate fertilizer on P content (%) in bulbs of the onion plant. Data on the left-hand side represent the 1st season and the right-hand side represent the 2nd season. Means with different letters for each group (Control or AMF) were considered statistically different.

Fig. 3 showed that P concentration in roots, shoots, and bulbs was increased in AMF inoculated plants compared to control. Relatively, AMF inoculation caused increments in P concentration in the root (Fig. 3a) by about 17% and 21% over the control for the first season and the second season, respectively. Moreover, AMF inoculation caused a relative increase in P concentration in the shoot (Fig. 3b) by about 29% and 17% over the control during the two seasons. P concentration in the bulb (Fig. 3c) almost followed the same trend as previously observed for root and shoot. P concentration in bulbs was considerably increased by inoculation with AMF during two seasons. P concentration in either root, shoot, or bulb reached maximum value when soil fertilizer with adding 120 kg P_2_O_5_ ha^−1^.

### 3.5 Effect of phosphorus fertilization and AMF inoculation on N-utilization by onion plant

Table 5 presented the effect of inoculation with AMF under different P levels on nitrogen utilization by onion. The obtained results clearly showed that AMF inoculation enhanced N utilization from chemical fertilizer more efficiently than those of the control (Without AMF). The highest value of N-utilized by the plant was detected with the full dose of 120 kg P_2_O_5_ ha^−1^ in presence of AMF inoculation during the two seasons. AMF inoculation caused increments in N utilization percentage by about 44.8, 47.8, and 23.0% for the 1st season and 24.1, 40.3, and 30.2% for the 2nd season over the control with three respective levels of P fertilizer. However, significant differences were obtained from the application at 80 and 120 kg ha^−1^ kg P_2_O_5_ ha^−1^. The values of N-utilized with AMF inoculation by onion plant as affected by the third level were nearly closed to those recorded with the second level through the second season.

**Table 5:** Effect of AMF inoculation on nitrogen utilization (%) of onion plant grown under different levels of P fertilizer.

P fertilizer conc. (kg ha^−1^)	Nitrogen utilization (%)			
	Season 2018/2019		Season 2019/2020	
	Control	AMF	Control	AMF
40	24.61	35.63	26.86	33.31
80	29.71	43.92	30.82	43.25
120	36.42	44.81	35.99	46.87
LSD (0.05)	P fertilizer: 2.17 AMF: 1.77 P fertilizer AMF: 3.07		P fertilizer: 2.42 AMF: 1.97 P fertilizer AMF: 3.42	

### 3.6 Effect of phosphorus fertilization and AMF inoculation on the economical yield

Results presented in Table 6 clearly showed that AMF inoculation and P fertilization had a significant effect on onion bulbs yield. During the two growing seasons, onion bulbs yield increased linearly with increasing phosphate fertilizer level with AMF inoculation. The increasing level of phosphate fertilizer progressively increased the economical yield of the onion plants across AMF inoculation (Table 6). Application of AMF inoculation with a level of 120 kg P_2_O_5_ ha^−1^ gave maximum values of economical yield were 32.69 and 32.12 ton ha^−1^ in the two seasons respectively but there no significant difference was obtained from application 80 and 120 kg P_2_O_5_ ha^−1^ during the two growing seasons.

**Table 6:** Effect of AMF inoculation on economical yield (bulb weight, ton ha^−1^) of onion plant grown under different levels of P fertilizer.

P fertilizer conc. (kg ha^−1^)	Economical yield (bulb weight, kg ha^−1^)			
	Season 2018/2019		Season 2019/2020	
	Control	AMF	Control	AMF
40	23,397	28,440	24,758	28,598
80	25,608	31,315	26,611	31,780
120	28,908	32,690	29,551	32,124
LSD (0.05)	P fertilizer: 842 AMF: 688 P fertilizer AMF: 1192		P fertilizer: 588 AMF: 480 P fertilizer AMF: 830	

## 4 Discussion

In the present study, the percentage of root colonization after 60 days with 80 kg P fertilizer ha^−1^ reached their maximal level of 70.0% with AMF inoculation followed by 120 and 40 kg P fertilizer ha^−1^. The highest AMF colonization percentage after 90 days with AMF inoculation reached 81.33% using 80 kg ha^−1^ P fertilizer. Alloush and Clark (2001) reported that AMF improves plant growth and nutrient uptake at low soil fertility and it can increase the effectiveness of P fertilizer added to soils than phosphate fertilizer added alone. Very low and very high phosphate fertilizer levels may reduce AMF infection colonization (Koide, 1991). Levels of soil P higher than that required for plant growth eliminated the development of the arbuscular of AMF. Arbuscules structure produced within the host plant cells by AMF resemble miniature shrub-like trees (Dickson et al., 2007), the addition of excess phosphate results in no arbuscules forming which are responsible for the transfer of absorbed nutrients from the fungus to the host plant (Hughes et al., 2008, Golubkina et al., 2018).

Our results showed that inoculation with AMF significantly increased available P in the rhizosphere compared to the control. Obtained results revealed that a reverse correlation can be drawn between P in the rhizosphere and mycorrhizal colonization. Our results further suggested that very high and very low available P levels in soil may reduce mycorrhizal colonization. These results are in agreement with those obtained by Koide and Li, 1990, Koide, 1991. The authors concluded that the level of P in the rhizosphere influence the establishment of mycorrhiza with a high-level inhibition of colonization. An increase in the level of P in soil results in a reduction in spores’ production by fungus these spores responsible for root infection (Menge et al., 1978). Smith and Read (2008) observed that P in soil is the most important edaphic item for the process of mycorrhizal symbiosis. Garcia and Mendoza (2008) reported that the best arbuscular colonization was related to the highest N and P concentrations in plant tissue, suggesting a correspondence with an increase in the rate of nutrient transfer between the cooperative accomplices.

In the current study, the fresh and dry weight, as well as total chlorophyll content of the onion plant, were linearly and positively affected by AMF inoculation. The maximum fresh and dry weights were on average observed under the highest phosphate level 120 kg P fertilizer ha^−1^. Similarly, Mohamed et al. (2014) reported that the total dry weight of the onion bulb gave maximum value when soil fertilized with NPK with inoculation by AMF. Mycorrhiza could increase all parameters because mycorrhizal fungi increase the absorption of water and mineral nutrient (Balloei et al., 2009, Golubkina et al., 2019). Also, Abadi et al. (2015) reported that the seed yield of the black cumin plant increased significantly with AMF inoculation. Moreover, AMF improved chlorophyll concentration and probably photosynthesis leading to increase dry mass bulb (Bolandnazar et al., 2007). Chanda et al. (2014) found that inoculation with AMF enchanted chlorophyll content compares to non-mycorrhizal. Also, Rahimi et al. (2012) found that the interaction of AMF inoculation and water stress created significant differences in chlorophyll *A*, *B*, and carotenoids. Singh et al. (2012) found that chlorophyll content increased after AMF inoculation as a result of higher concentrations of Mg, Fe, and Cu tissues influencing chlorophyll synthesis.

Maximum P concentrations in roots, shoots, and bulbs were increased compared to control due to AMF inoculation. In the same connection, Karandashov and Bucher (2005) reported that one of the most effects of AMF inoculation on the host plant is the increase in P in the plant mainly due to mycorrhiza absorbed phosphate from the soil and transfer it to host roots. The fungal hyphae transport phosphate large distances into roots cortical cell (Parkash and Aggarwal, 2009, Golubkina et al., 2020b). Clark and Zeto (2000) demonstrated that AMF plays a dominant role in increasing phosphorus solubilization and nutrient uptake as P, N and K. Podila and Douds (2010) reported that AMF improved absorption of P and other nutrients by plants.

Nitrogen is a major essential element and the amount of available nitrogen in the soil is a limiting factor for natural and agricultural plant production (Ohyama et al., 2014). Our results showed that N-fertilizer was more efficiently used by inoculated plants as compared to control. ^15^N dilution techniques have been decisive for evaluating the impact of agronomic management on N and P cycling. Stable isotope label has suggested that inorganic nitrogen is taken up by the extra mycelium, incorporated into amino acids, translocated from extra to intra-mycelium fungal structures as arginine, and then transported as ammonium to the plant (Govindarajulu et al., 2005, Jin et al., 2005).

In this study, the highest bulb weight was found in response to the application of 80 kg ha^−1^ P fertilizer due to the adequate nutrient, moisture, and light which helped to increase the average weight of bulb per plant (Khan et al. 2002). In the same connection, onion response to the phosphate fertilizer application of up to 90 kg ha^−1^ P fertilizer was reported by Costa et al. (2009), obtaining a yield of 33.4 kg ha^−1^. Resende et al. (2016) observed maximum yields for two onion cultivars with a dose of 132 kg ha^−1^ P fertilizer, comparable to the economic dose of 130 kg ha^−1^ P fertilizer. The application of 71 kg ha^−1^ of P fertilizer provided the commercial maximum yield of bulbs in onion crop, as well as 60 kg ha^−1^ P fertilizer by Wamser et al. (2011) and 132 kg ha^−1^ P fertilizer by Resende et al. (2016).

## 5 Conclusion

This study indicated that AMF can increase the production of onion yield with second and third levels from P fertilizer under drip irrigation during two successive seasons. Inoculating onion plant in the field with AMF could be effective onion plant production as a result of the fertility root zone which can enhance absorption of nutrients and then improved yield recommend before inoculating soil with AMF. Economic onion yield (dry bulb weight) increased significantly with increasing P fertilizer 120 kg ha^−1^ by AMF inoculation. These findings suggested that AMF will be, in the near future, an emerging and promising candidate in sustainable agriculture, as alternative green technology to minimize the use of synthetic fertilizers.

## Declaration of Competing Interest

The authors declare that they have no known competing financial interests or personal relationships that could have appeared to influence the work reported in this paper.
